# Supplementary material for: Identification and characterisation of the CD40-ligand of Sigmodon hispidus
Source: PLoS One. 2018 Jul 27;13(7):e0199067. doi: 10.1371/journal.pone.0199067 (PMC6063397; doi:10.1371/journal.pone.0199067)
Supplement: S1 Fig — (PDF) [file pone.0199067.s001.pdf]

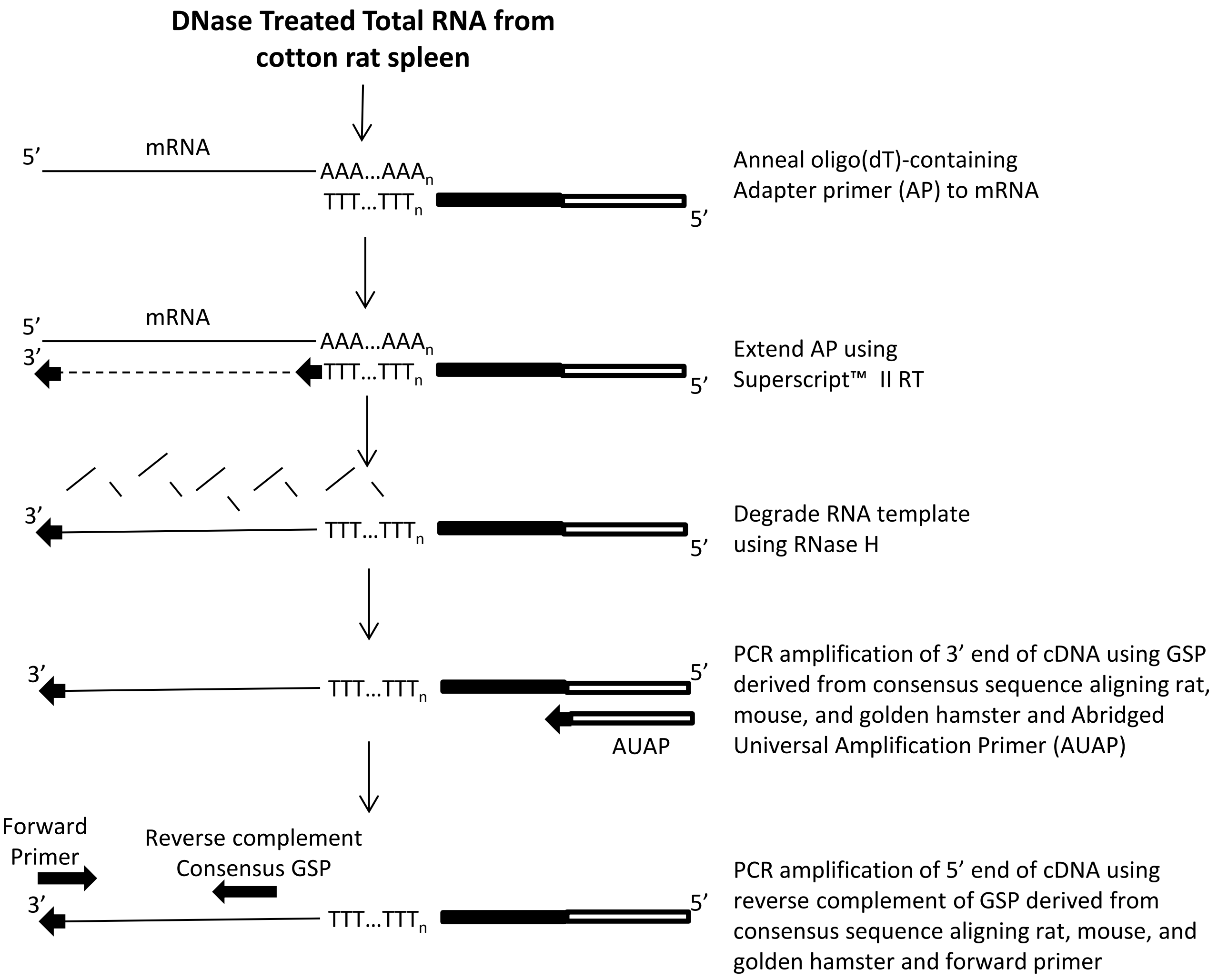

S1 Figure: Summary of the 3'RACE strategy used to elucidate the Cotton Rat CD40L gene sequence. The 3' RACE utilizes the poly(A) tail region as an initial priming site and a gene specific primer (GSP) derived from a consensus sequence aligning the rat, mouse, and golden hamster CD40L gene sequences. The reverse complement of this same primer was used to amplify the 5' end of the Cotton Rat CD40L cDNA sequence with a forward primer.
