# Supplementary material for: Identification and characterisation of the CD40-ligand of Sigmodon hispidus
Source: PLoS One. 2018 Jul 27;13(7):e0199067. doi: 10.1371/journal.pone.0199067 (PMC6063397; doi:10.1371/journal.pone.0199067)
Supplement: S2 Fig — (PDF) [file pone.0199067.s002.pdf]

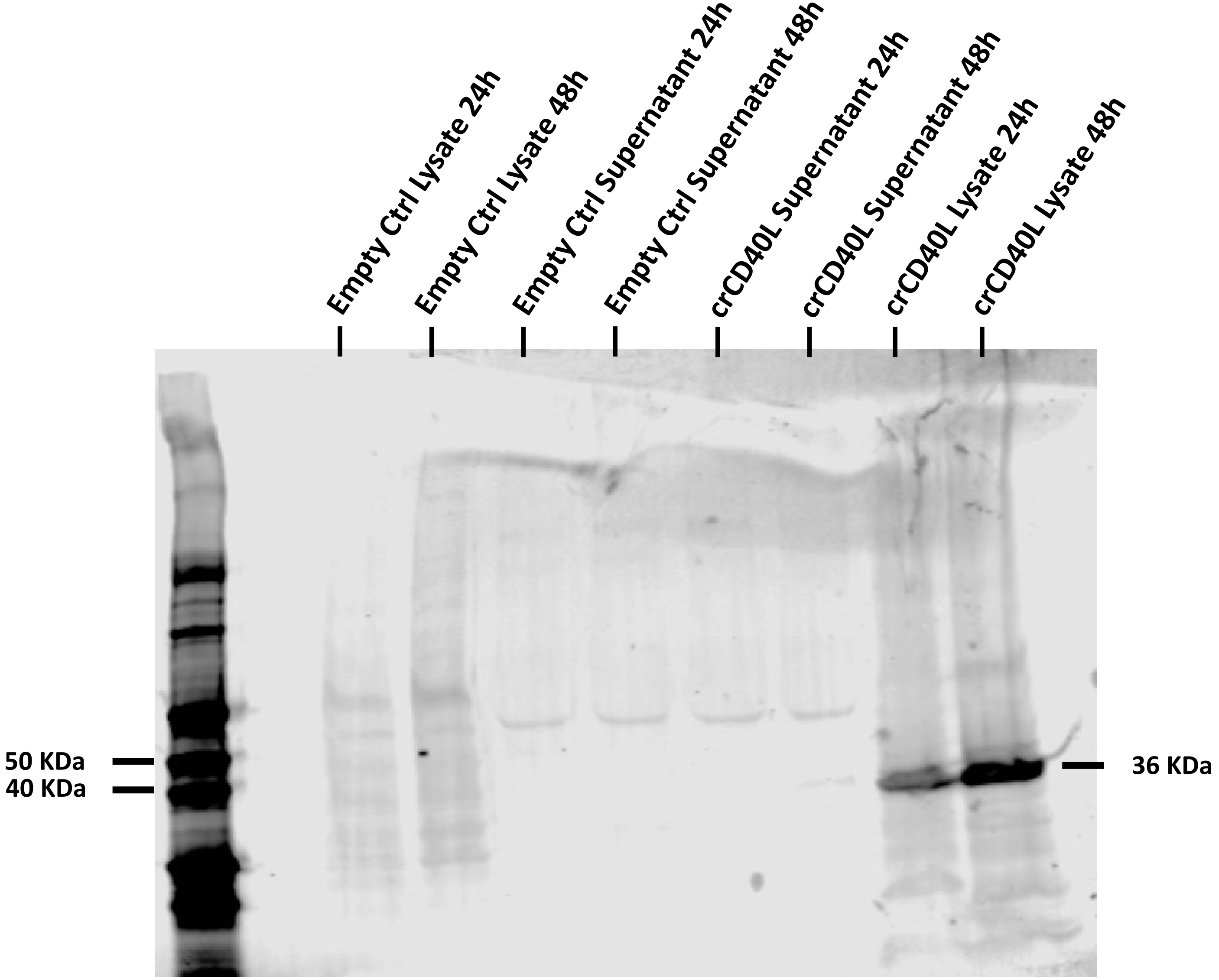

S2 Figure: crCD40L construct and protein expression and secretion. In vitro protein expression in BHK21 cells and supernatant collected 24h and 48h post infection. Protein expression is confirmed by Western blot using an anti-HIS antibody.
