# Supplementary figures and images for: Identification and characterisation of the CD40-ligand of Sigmodon hispidus
Source: PLoS One. 2018 Jul 27;13(7):e0199067. doi: 10.1371/journal.pone.0199067 (PMC6063397; doi:10.1371/journal.pone.0199067)

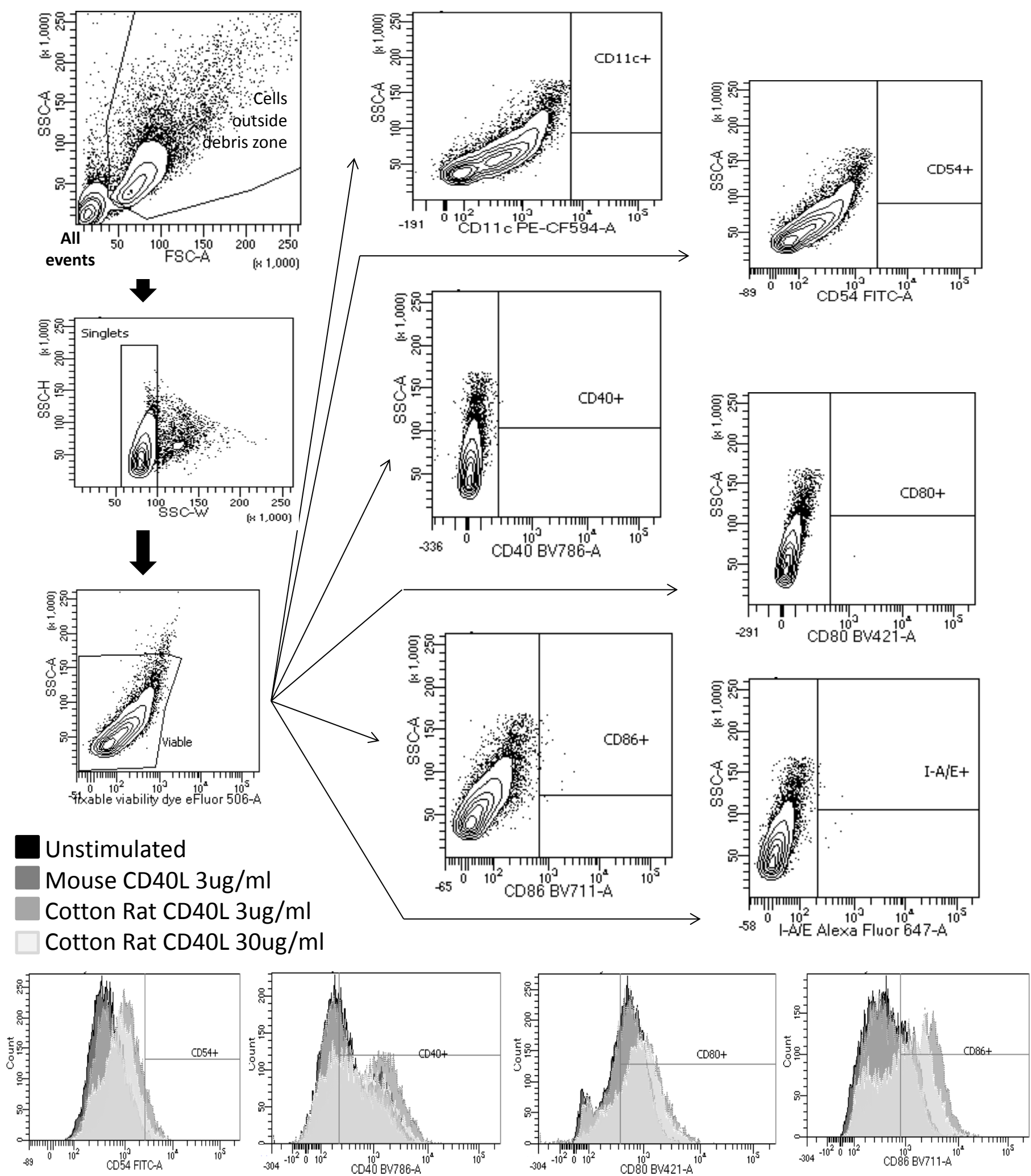

**S3\_Figure: Gating strategy for flow cytometry analysis of dendritic cells.**

Supplement: S3 Fig — (PDF) [file pone.0199067.s003.pdf]
